# γδT cells in oral tissue immune surveillance and pathology

**DOI:** 10.3389/fimmu.2022.1050030

**Published:** 2023-01-10

**Authors:** Yilong Chen, Juan Du, Yitong Liu, Zhenhua Luo, Lijia Guo, Junji Xu, Lu Jia, Yi Liu

**Affiliations:** ^1^ Laboratory of Tissue Regeneration and Immunology and Department of Periodontics, School of Stomatology, Capital Medical University, Beijing, China; ^2^ Beijing Key Laboratory of Tooth Regeneration and Function Reconstruction, School of Stomatology, Capital Medical University, Beijing, China; ^3^ Department of Orthodontics School of Stomatology, Capital Medical University, Beijing, China

**Keywords:** γδT cells, oral mucosa, microbiota, periodontitis, bone remodeling

## Abstract

The oral mucosa’s immune system is composed of tissue-resident and specifically recruited leukocytes that could effectively tolerate a wide range of microbial and mechanical assaults. Shortly after CD4^+^ helper T cells (TH17 cells) that produce interleukin 17 (IL-17) were identified, it was discovered that γδT cells could also induce substantial levels of this pro-inflammatory cytokine. In the past decades, it has become clear that due to a complicated thymic program of development, γδT cells frequently serve as the primary sources of IL-17 in numerous models of inflammatory diseases while also assisting in the maintenance of tissue homeostasis in the skin and intestine. But it wasn’t until recently that we took thorough insight into the complex features of γδT cells in the oral mucosa. Most gingival intraepithelial γδT cells reside in the junctional epithelium adjacent to the dental biofilm, suggesting their potential role in regulating oral microbiota. However, inconsistent results have been published in this regard. Similarly, recent findings showed contradictory data about the role of γδT lymphocytes in experimental periodontitis based on different models. In addition, conflicting findings were presented in terms of alveolar bone physiology and pathology underlying the oral mucosa. This review provided an overview of current knowledge and viewpoints regarding the complex roles played by oral-resident γδT cells in host-microbiota interactions, gingivitis and periodontitis, bone physiology and pathology.

## 1 Introduction

**Figure 1:**
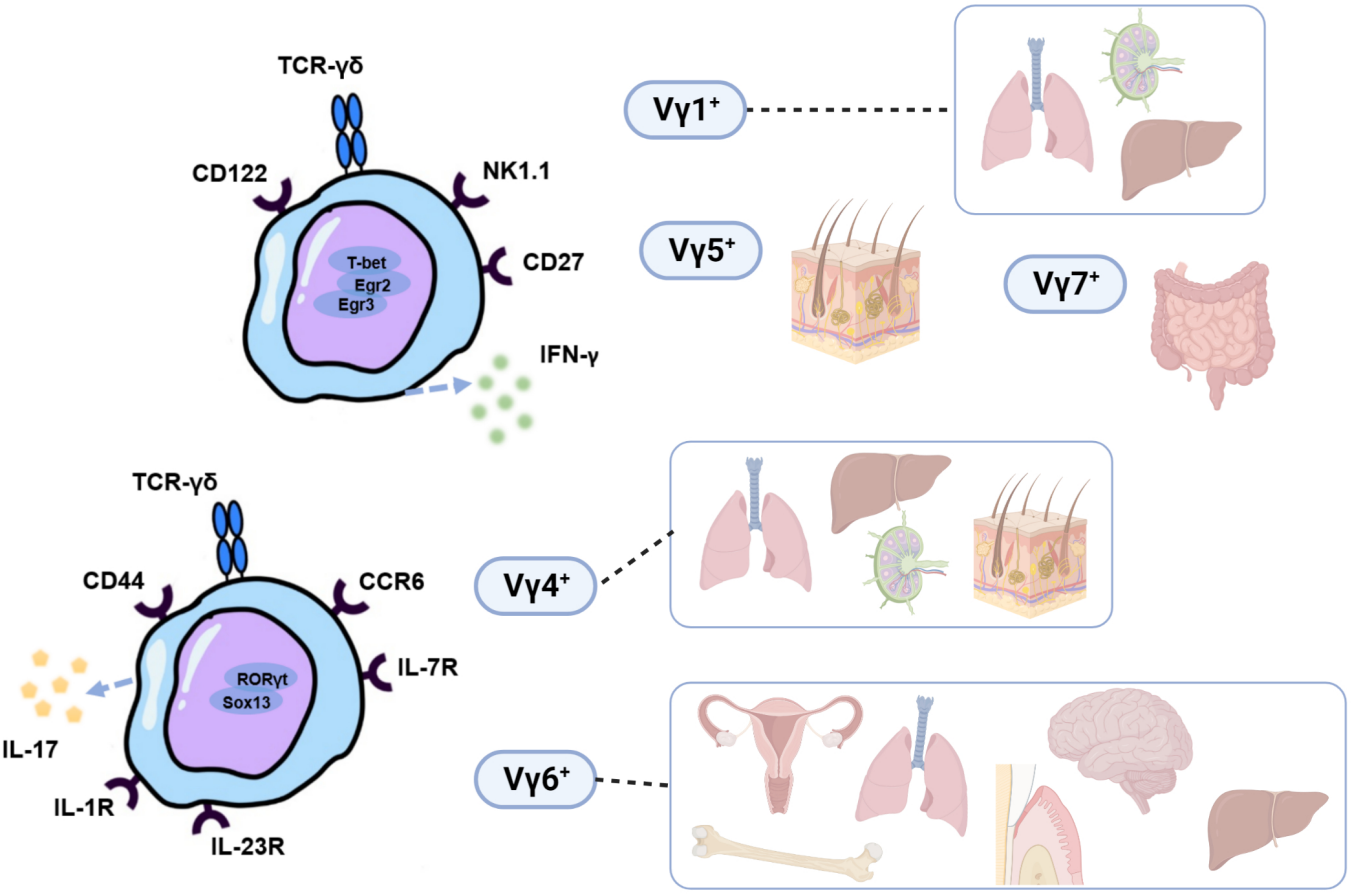
Functional and distributional characterization of murine Vγ1^+^, Vγ4^+^, Vγ5^+^, Vγ6^+^, and Vγ7^+^ γδT cells subsets. IL-17 production is mainly restricted to Vγ4^+^ or Vγ6^+^ γδT cell subsets while Vγ1^+^, Vγ5^+^, and Vγ7^+^ γδT cells are associated with the secretion of IFN-γ. Different surface receptors that IL-17+and IFN-γ+ γδT cell subsets express allow for their identification and isolation. γδ17T cells express IL-1R, IL-23R, IL-7R, CCR2, CCR6, and CD44 on the cell surface, and the transcriptional factors of RORγt, Blk, Hes-1, Sox4, and Sox13 are important for γδ17T cells development and function. IFN-γ-producing γδT cells, on the other hand, exhibit cell surface markers CD27, CD122, and NK1.1 (8). They selectively home to various organs, and serve important functions in tissue immune surveillance and homeostasis.

γδT cells are a special type of lymphocyte that participate in innate and adaptive immune responses (1). Different subsets of γδT cells can be innate-like (2), adaptive (3) or share the characteristic of both (4, 5). They develop in the embryonic and postnatal thymus, conserved among almost all jawed vertebrates, including mice and humans (6). Unlike αβT cells and B cells, γδT cells make up a small proportion of lymphocytes in the blood and secondary lymphoid organs, but they are found to be more prevalent in peripheral epithelial sites, such as mucosal tissues and skin (7). After developing in the thymus (8), they exit it in waves of cells that express various γδTCRs and reside in peripheral tissues (as reviewed by Papotto et al. (9)). Based on the V-segment that γδT cells express in the variable area of mice γδT cell receptor (TCR) -chain, γδT cells can be categorized into many subgroups, namely Vγ1^+^, Vγ4^+^, Vγ5^+^, Vγ6^+^, and Vγ7^+^ γδT cells (10). These subgroups have varied tissue localization, release of unique cytokines, and go through several stages of thymic development (11, 12). Vγ5^+^ and Vγ6^+^ γδT cells emerge as natural effector T cells solely in the embryonic thymus and have a limited TCR repertoire, in contrast to Vγ1^+^ and Vγ4^+^ γδT cells, which arise from both adult and embryonic thymus. In particular, IL-17 production is mainly restricted to Vγ4^+^ or Vγ6^+^ γδT cell subsets. On the other hand, Vγ1^+^, Vγ5^+^, and Vγ7^+^ γδT cells are associated with the secretion of IFN-γ (9), although Vγ1^+^, Vγ5^+^γδT cells can also produce IL-17 in some cases (13, 14) (**Figure 1**). Depending on the expression of TCRδ chains and cell function, human γδT cells can be briefly separated into the subpopulations of Vδ1^+^, Vδ2^+^, and Vδ3^+^ γδT cells (15). The most abundant human γδT cells are Vγ9Vδ2T cells; they consist of nearly 98% of human peripheral blood γδT cells (16) and are activated primarily by non-protein pyrophosphate metabolites phosphoantigen (17). They have been verified to have potent anti-tumor activity, implying their promising usage in clinical treatments for patients with malignant tumors (1, 18, 19). Vδ1^+^ and Vδ3^+^γδT cells, on the other hand, are more common in peripheral tissues (20). Most Vδ1^+^γδT cells are found in the epithelial tissues, for instance, the intestine and the skin. They could recognize infected and cancerous cells and function in immune response with cytotoxic capability and anti-cancer activity (21). Vδ3^+^γδT cells are primarily found in the liver and intestines and are implicated in response to various virus infections, whereas their functions need further investigation (15).

In recent years, various studies revealed that the ability to produce IL-17 is critical for both immunopathology and host resistance to certain pathogens (22–25). It has also been confirmed that interleukin-17A (IL17A), which is secreted by various innate and adaptive immune cells, can mediate the occurrence and progression of periodontitis (26). γδ17T cells are one of the primary sources of IL-17 in mice and humans (27–29); by rapidly producing large amounts of IL-17, γδ17T cells are discovered to defend the body surface in several epidermal and mucosal areas (30–33) from bacterial (34), fungal (35), and malarial infections (36). In addition to their immune surveillance activities, recent reports have unraveled exciting new roles for γδ17T cells in steady-state tissue physiology, with functions ranging from regulating thermogenesis in adipose tissue (37) to repairing epithelial and mucosal barriers (38). Notably, a current study elucidated the crucial role of γδ17T cells in the hypoxic adaption of wound-edge epithelium *via* Interleukin-17 signaling (39).

As reviewed previously, the activation of γδ17T cells in inflammation was mostly *via* the production of IL-1β and IL-23 by DCs and macrophages (40) in response to the presence of pathogen-associated molecular patterns and can result in cell expansion, increased IL-17 production, and recruitment of neutrophils (9). It has also been identified that γδT17 cells can proliferate by the selective promotion of IL-7 (41). Moreover, it has been shown that IL-17-producing γδT cells expressed Toll-like receptors TLR1 and TLR2 and could directly interact with certain pathogens, representing an innate protective response to bacterial infection *in vitro* (42). Interestingly, a recent study revealed a novel mechanism where γδT17 cells are regulated by microbiota dysbiosis through cell-to-cell contact with activated CD103^+^CD11c^+^DCs (43). These DCs were discovered to reside with γδT17 cells in stratified squamous epithelia of skin and mucosa and were found to be regulated *via* sequential bone morphogenetic protein 7 (BMP7) and TGF-β1 signaling in a microbiota-dependent manner (44). These findings collectively demonstrate that cytokine and certain pathogens stimulation can promote the growth and proliferation of γδ17T cells. (**Figure 1**)

While most research on intraepithelial γδT cells focuses on skin and intestine epithelia, our understanding of these cells in the oral mucosa remains limited. Only recently did studies shed light on the unique role of γδT cells in terms of homeostasis maintenance, periodontitis, and bone remodeling in the oral environment. We will outline here the current understanding and outstanding questions regarding those issues.

## 2 The interplay between γδ17T cells and microbiota in oral mucosa

Oral epithelial tissues line the oral cavity and effectively act as a barrier to the external environment (45). The oral epithelium, which is in direct contact with the outside environment, offers vital protection against constant physical stress (46) and pathogen invasion (47–50). The uniqueness of the oral cavity also lies in the presence of a specialized interface between hard and soft tissue (51). Therefore, it is necessary to pose strict management of tissue integrity and epithelial immune surveillance. In epithelial barrier tissues, various pieces of evidence have elucidated the role of IL-17+γδT cells as local sentinels and mediators of host-microbial homeostasis, including skin (52), intestine (53), testicle (54), and vagina (55). As for the oral environment, evidence displayed that oral bacterial colonization may influence the development and maintenance of γδ17T cells. The other way round, it was found that most gingival intraepithelial γδT cells reside in the junctional epithelium, where they are adjacent to dental biofilm, implying their possible role in the host-microbiota interactions in the gingiva (56).

**Figure 2:**
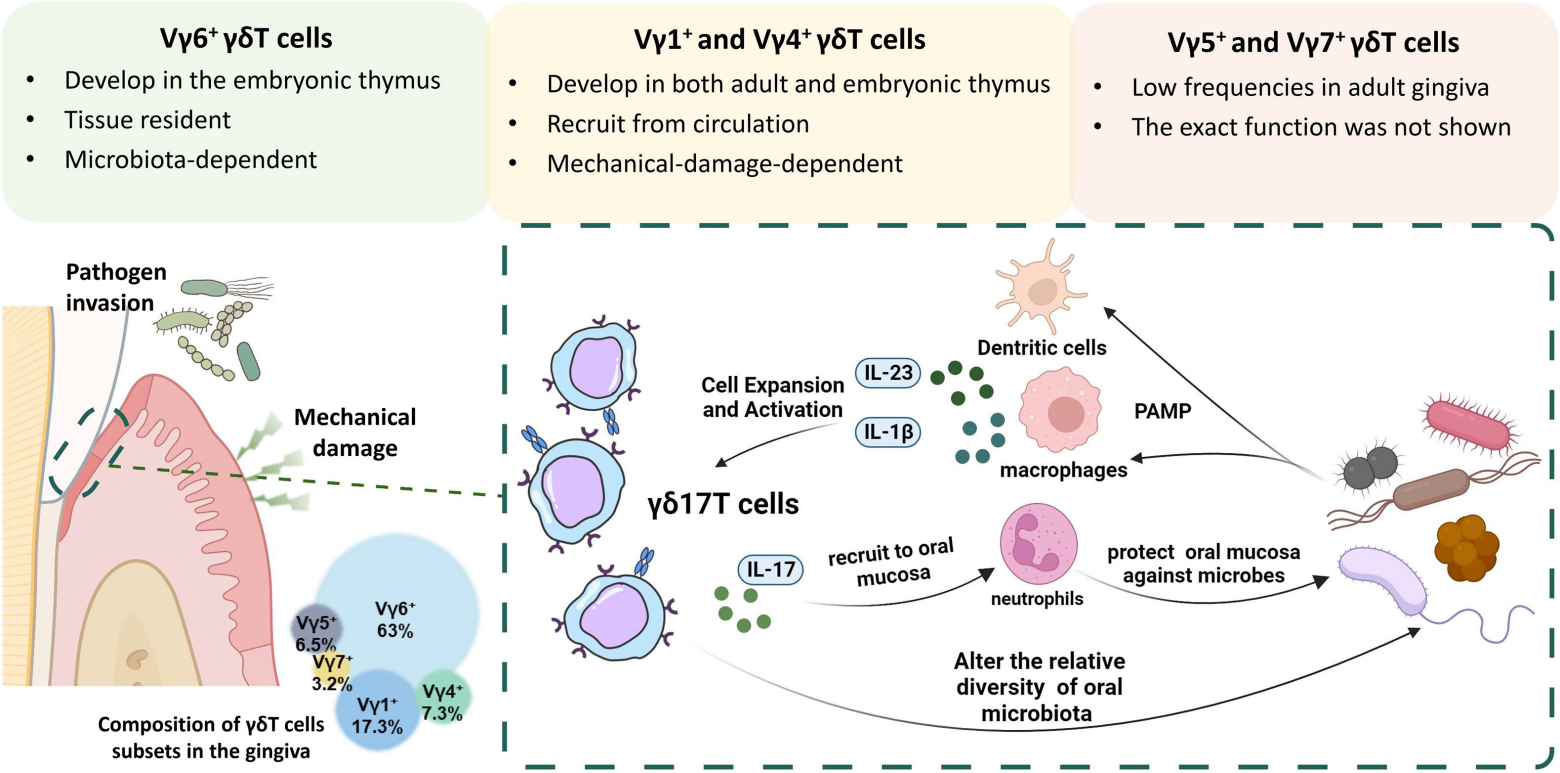
The interplay between γδ17T cells and microbiota in the oral mucosa. Gingival γδT cells are mainly located in the junctional epithelium and the lamina propria and are primarily composed of Vγ6^+^, Vγ1^+^, and Vγ4^+^ subsets. The Vγ5^+^ and Vγ7^+^ subgroups can also be found in the gingiva, albeit at much lower frequencies. During embryogenesis, Vγ6^+^ and Vγ5^+^ γδT cells develop in the embryonic thymus and reach the oral mucosa during embryogenesis, while Vγ1^+^ and Vγ4^+^ develop in both embryonic and adult thymus. Over the suckling and weaning period, the Vγ6^+^ subset expands locally in a microbiota-dependent manner, whereas the Vγ4^+^ and Vγ1^+^ subsets arrive at the oral mucosa *via* circulation. Most mice gingival γδT cells express Vγ6^+^ TCR-chain and have a preactivated phenotype representing the main IL-17–producing lymphocyte subset in oral tissues. Under the constant challenge of physical stress and pathogen invasion, specific cytokines and pathogens stimulation can promote the growth and proliferation of γδ17T cells. Activated γδ17T cells then recruit neutrophils to the junctional epithelial site by IL-17 signaling and protect against oral pathogens.

Gingival γδT cells of adult mice are composed of Vγ6^+^ (~63%), Vγ1^+^ (~17%), Vγ4^+^ (~7%), Vγ5^+^ (~6%), and Vγ7^+^ (~3%) subsets (**Figure 2**). Most mice gingival γδT cells express Vγ6^+^ TCR-chain and have a preactivated phenotype representing the main IL-17–producing lymphocyte subset in oral tissues. They enter the oral epithelium during embryogenesis, reside mainly at the junctional epithelium, and increase during suckling and weaning. Later, a gradual loss of Vγ6^+^γδT cells in mice was observed with aging (56).

As mentioned above, Vγ6^+^ γδ17T cells can be activated by various signaling in a microbiota-dependent manner. Germ-free (GF) mice were used to investigate whether oral microbiota has an impact on γδT cells. It was discovered using GF mice that the lack of the microbiota led to reduced frequencies and overall numbers of gingival γδT lymphocytes, especially the Vγ6^+^ subset. Furthermore, adult mice treated with antibiotics had a substantial decrease in the frequency of intraepithelial γδT cells in their gingiva (56), indicating that the microbiota may have an impact on the development and maintenance of these cells. However, the number of Vγ4^+^ subsets and αβT cells remained relatively unchanged in the GF mice model (56). Furthermore, it was discovered that, rather than an alteration in microbiota, chronic elevation in mechanical barrier damage would result in increased recruitment of Vγ1^+^ and Vγ4^+^ subsets (57). And therefore, gingival Vγ6^+^ γδ17T cells appeared to be the most related to changes in oral microbiota (**Figure 2**).

**Figure 3:**
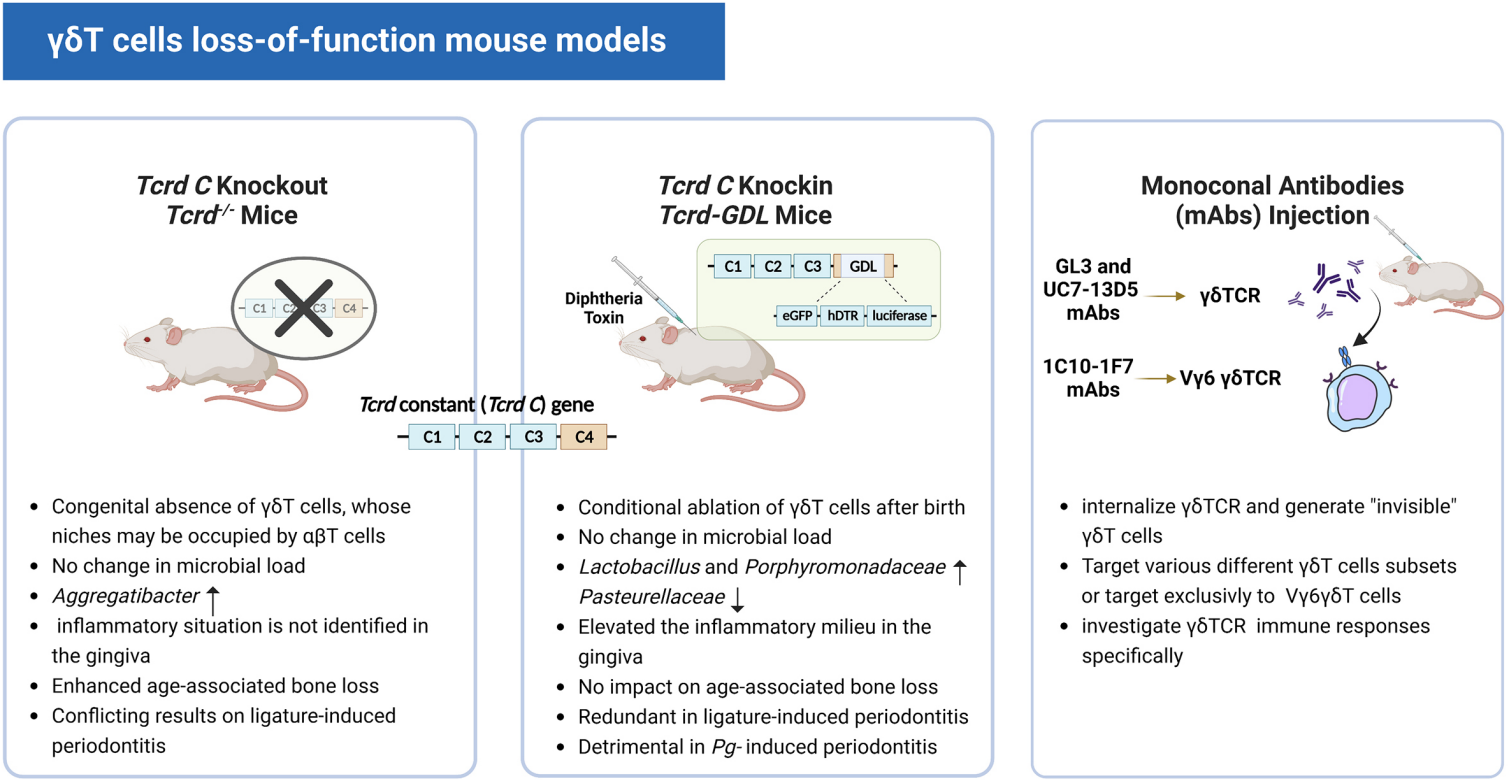
Three different γδT cell loss-of-function mouse models. The images show the characteristics of the three different mouse models and their respective relevant experimental results.

To better understand the role of γδT cells, in the early 90s, congenital genetic ablation of γδT cells arose for loss-of-function studies (58, 59). However, recent research revealed that αβT cells could occupy the absent γδT cells’ niches and possibly take over some of their functions in *Tcrd^-/-^* mice whose γδT cells are absent from birth (60). Sandrock et al. built a brand-new *Tcrd-GDL* knock-in mice model that expresses three reporter genes G-D-L, GFP, diphtheria toxin (DT) receptor, and luciferase concurrently. When the DT is injected into Tcrd-GDL mice, the expression of the DT receptor allows for *in vivo* γδT cell conditional depletion (27). Notably, IFN-γ+ γδT cells gradually reappear within two weeks after DT treatment and are fully restored within seven weeks. The γδ17T cells, on the other hand, remain at relatively low frequencies even after seven weeks of DT treatment. This *Tcrd-GDL* knock-in mice model provided us with a novel method to investigate the interaction between γδ17T cells and the oral microbiota while leaving the establishment of oral mucosa homeostasis intact. However, for more in-depth studies, depletion for a longer period of time may be required, since this model only provides temporary depletion, repeated injections of DT over time are required. Previous studies reported that this caused no abnormalities in wild-type mice (61) and had no effect on gingival immunity or pathology (44, 62). Wilharm et al. detected an increase in neutrophils, monocytes, and FOXP3^+^CD4^+^ T cells (T regulatory cells) after 5 months of depletion with the injection of DT on a weekly basis (56). Therefore, concerning the repeated injection of DT and its likely impact on immunoreaction, this model still has its shortcomings for the study of a long time span. (**Figure 3**) Furthermore, in addition to the genetic depletion of γδT cells, other strategies for depleting γδT cells *in vivo* use monoclonal antibodies (mAbs), such as GL3 (63, 64) and UC7-13D5 (65–67) antibodies targeting γδTCR. Notably, a new mAb 1C10-1F7 specific to the mouse Vγ6 chain was developed recently (68). Instead of depleting γδT cells, these attempts turned out to internalize TCR and generate “invisible” γδT cells (69) and may serve as a functional ablation (70, 71). In this context, γδT cells with no detectable TCR levels on their cell surface may respond poorly to TCR-specific stimulation. Therefore, it is undoubtedly helpful to investigate the specific role of the γδTCR in the immune responses of γδT cells, such as in malaria (36). However, such a γδT-less system might be inadequate to study the function of γδT cells in a TCR-independent manner, for example *via* cytokine receptors or Toll-like receptors (**Figure 3**)

**Figure 4:**
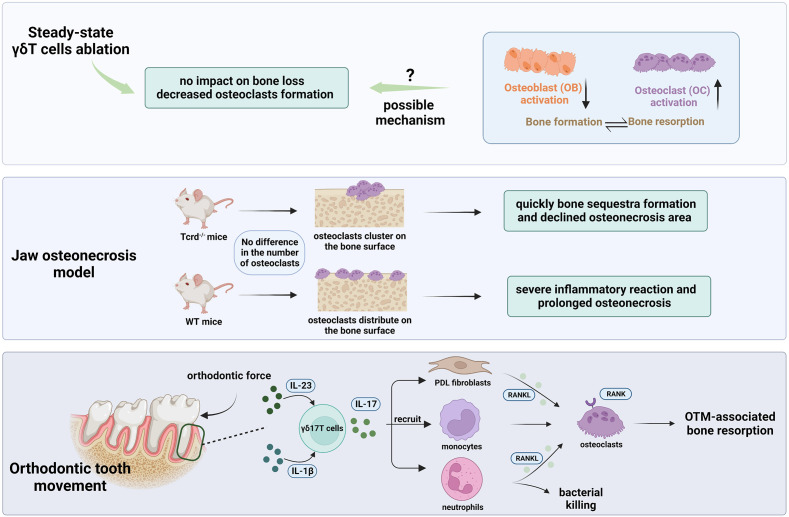
The role of γδ17T cells in oral bone physiology and pathology. γδT cell depletion in steady-state using Tcrd-GDL mice (Top bar). No increased alveolar bone loss was discovered after the DT injection. However, a decline in osteoclast levels around the alveolar bone was detected implying the likely function of γδT cells in controlling osteoclast formation. The absence of an overall effect on alveolar bone may be result from the suppression of osteoblasts activity or a greater activation of osteoclasts. In the jaw osteonecrosis model (middle bar), instead of quantity, γδT cells have an impact on the osteoclasts’ distribution on the palatal surface after tooth extraction. In the movement of orthodontic teeth (bottom bar), as the major source of IL-17A in the PDL, γδT cells could upregulate RANKL expression by PDL fibroblasts (103), recruit neutrophil and monocytes, and therefore facilitate osteoclast formation and subsequently bone resorption in the pressure site during OTM.

Using γδT cells loss-of-function models, it has been confirmed that the absence of γδ17T cells has an impact on oral microbiota in steady-state. Conditional ablation of γδT cells using *Tcrd-GDL* mice could lead to oral microbial dysregulation, with *Lactobacillus* and *Porphyromonadaceae* species growing, *Pasteurellaceae* species declining, but *Streptococcaceae* species remaining unaffected (56). It’s worth noting that similar microbial alterations were observed in IL17R-deficient animals (43, 72, 73). As the primary IL-17-producing cells in the oral mucosa (56), this emphasizes the significance of steady-state IL-17 signaling by γδ17T cells in the adult phase. In line with these findings, based on a more recent study, γδ17T cells are involved in the immunological and functional processes right after birth, in response to the initial contact with the microbiota that occurs in the oral epithelium (74). Using *Tcrd-GDL* mice, it was confirmed that Vγ6^+^ γδ17T cells play a vital role in the recruitment of neutrophils to the neonatal buccal and tongue epithelium by producing IL-17 in a microbiota-dependent manner. And these recruited neutrophils in the neonatal oral epithelia are likely to offer protection against exposure to high microbial loads since the neonatal epithelia are hyperpermeable and momentarily vulnerable compared to adult ones. Of note, >90% of the IL-17-producing cells in the neonatal oral epithelium are γδ17T cells (74). In contrast, the rate has been reported to be about 70% in steady-state gingiva of adult mice (56). Thus, these findings indicate their potential role in the surveillance and establishment of oral microbiota in the neonatal phase. However, only an expansion of *Aggregatibacter* species was shown using *Tcrd^-/-^* mice in terms of the load and taxon richness of the oral microbiota (57). (**Figure 2**) The oral microbiota may be a significant contributor to such conflicting outcomes since *Tcrd^-/-^* mice are born without γδT cells, which were discovered to be crucial in the postnatal development of oral host-microbiota homeostasis (74), and this parallels the key difference between *Tcrd^-/-^* and *Tcrd-GDL* mice in terms of the interplay of γδT cells and the oral microbiota.

## 3 The role of γδ17T cells in gingivitis and periodontitis

Awareness of the role of γδ17T cells in periodontitis is not recent, having possibly first been described in the early 90s. In both gingivitis and periodontitis tissues, a rising proportion of γδT cells with increasing size of infiltration has been demonstrated (75). Lundqvist’s findings implied that γδ17T cells serve as the first line of defense in the inflamed gingiva, blocking the entry of pathogens through cytotoxicity against infected and stressed epithelial cells and by regulating epithelial cell development through the release of regulatory cytokines (76). Moreover, abnormal proportions of γδT cells have also been detected in the peripheral blood of patients with periodontal disease (77).

To directly study the role of γδ17T cells, *Tcrd^-/-^* mice missing γδT cells from birth were given ligature-induced periodontitis, yet the findings were inconsistent. A study by Krishnan et al. revealed that bone loss was increased in *Tcrd^-/-^* mice compared to WT mice after ten days of inflammation (57). This is in line with the previous study that reported protective roles for IL-17 in periodontitis (78). Nevertheless, Tsukasaki et al. could not detect any difference between the two genotypes at that time (79). They also found that, while the number of γδT cells was unaffected, Foxp3^+^ T regulatory cells, which were transformed into Th17 cells, proliferated in the periodontal lesion and caused bone loss.

Likewise, recent studies using the *Tcrd-GDL* mice and ligature-induced periodontitis model showed that the IL-17 production by CD4^+^ αβT cells (Th17 cells) in the gingiva appeared to be more vital than that of γδ17T cells in terms of the pathology of experimental periodontitis (80, 81). Such conflicting reports on the role of γδT cells in ligature-induced periodontitis may result from technical variations in ligature placement. And again, this is consistent with the fundamental distinction between *Tcrd^-/-^* and *Tcrd-GDL* mice in terms of how γδT cells affect the oral microbiota.

However, although IL-17 was documented to mediate periodontal damage (80), it is enigmatical why γδ17T cells, the largest producers of IL-17 in the steady-state gingiva (56), are unnecessary during ligature-induced periodontitis. Other than the gingiva, IL-17 production by γδ17T cells in epidermis and epithelium tissues are significantly linked to inflammation-exacerbation and tissue-damaging side effects, such as psoriasis (82, 83) or airway inflammations (84, 85). One possible explanation is that gingival Th17 cells react more quickly to a shift in the microbiota (80) and gingival γδT cells are more focused on preserving tissue homeostasis and repairing the damage (38). Given that the Vγ6^+^ γδT cells subset, which constitutes the majority of T cells in adult gingiva, has an embryonic origin, it is consistent with this notion that these cells populate the epithelium prior to exposure to the oral microbiota.

Of late, a study by Bare et al. may give us some novel insights into the unique role of γδ17T cells in periodontitis (86). Although their findings identified again that γδ17T cells are not necessary for bone loss brought on by the ligature-induced periodontitis, they surprisingly found that γδ17T cells promote periodontal damage driven on by oral infection with *Porphyromonas gingivalis*. Conditional ablation of γδT cells prevented *Pg*-induced osteoclast genesis and diminished the recruitment of neutrophils as well after oral infection, in line with the decrease of bone loss observed in these mice. (**Table 1**) Worthy of attention, a previous study found that in the circumstances of continuous mechanical stimulation, Th17 cells would accumulate in a way that is independent of commensals but dependent on IL-6, which is mainly secreted by epithelial cells (46). In this view, when a significant amount of tissue stress occurs during ligature installation, Th17 cells may start to accumulate at the epithelial site predominantly and have a more substantial role in the pathology of ligature-induced periodontitis. While on the other hand, γδT cells may trigger a tissue repair response (57) that prevents excessive bone loss induced by Th17 cells.

**Table 1:** The role of γδ17T cells in gingivitis and periodontitis.

Materials/models		Findings	References
Clinical Samples			
**Peripheral blood from 16 periodontitis patients**		Increased proportions of γδT cells in the peripheral blood of patients with periodontal disease	(77)
**Gingival tissue obtained from 55 patients**		γδ17T cells can be activated in periodontitis gingiva and serve as the first line of defense in the inflamed gingiva	(76)
**Gingival tissue obtained from 32 patients**		An increasing proportion of γδT cells in infiltration site	(75)
Animal Experimentation			
**Ligature-induced periodontitis**	*Tcrd^-/-^* mice	γδ17T cells play a protective role in periodontitis	(57)
		γδ17T cells are dispensable for periodontitis pathology	(79)
	N/A	Instead of γδ17T cells, Th17 cell-derived IL-17 mediates pathology in periodontitis	(80)
	*Tcrd^-/-^* mice and *Tcrd-GDL* mice	γδ17T cells are dispensable for periodontitis pathology in a medium timespan (four weeks)	(81)
	*Tcrd-GDL* mice	γδ17T cells are not necessary for bone loss in periodontitis	(86)
***Pg*-induced periodontitis**	*Tcrd-GDL* mice	γδ17T cells are essential to bone loss induced by oral infection with *Pg*	

## 4 The role of γδ17T cells in oral bone physiology and pathology

T-lymphocytes and bone physiology have long been known to be strongly connected (87–89). However, the role of IL-17 and γδT cells in oral bone physiology and pathology remains contradictory (90, 91).

Ono et al. were the first to establish that murine Vγ6^+^ γδT cells were the primary source of IL-17A in the process of bone regeneration in a drill-hole injury model. IL-17A is enormously elevated after bone injury and stimulates osteoblast genesis of mesenchymal cells from the repair tissue (92). *In vitro* research using phosphoantigen-activated human γδT cells isolated from hPBMC (Vγ9Vδ2 T cells), it was verified that γδT cells could inhibit osteoclasts generation and function from monocyte precursors (93). Moreover, Vγ9Vδ2T cells stimulated by zoledronate acid can inhibit immature dendritic cells (iDCs) transdifferentiation into osteoclasts by downregulating RANK *in vitro* (94). In addition, it was found that Vγ9Vδ2T cells activated by phosphoantigen can transform into effector memory cells (TEM), and secrete a relatively high level of IL-17 and IFN-γ. Compared to the freshly isolated γδT cells from hPBMC, they are able to secrete IFN-γ solely (95). This finding provided us with fresh clues to explore the mechanism of how human γδT cells may have an impact on bone physiology.

In the oral environment, Yu et al. discovered that mice lacking the IL-17A receptor (IL17AR) exhibit accelerated alveolar bone loss in this periodontitis model, indicating their protective role in bone destruction (78). The induction of IL-17 and amphiregulin by γδT cells has been demonstrated to have a significant role in protecting against age-associated periodontal bone loss in *Tcrd^-/-^* mice (57), which is in line with the previous findings. Contrarily, it was noted that the RANKL/Osteoprotegerin (OPG) ratio did not change after γδT cell depletion in steady-state. As a result, no evidence of increased alveolar bone loss was discovered in *Tcrd-GDL* mice five months after DT injection (56). Additionally, they discovered a decline in osteoclast levels around the alveolar bone as early as two weeks following the ablation of γδT cells, implying the likely function of γδT cells in controlling osteoclast formation. The absence of an overall effect on alveolar bone may indicate that osteoblasts are affected by the loss of γδT cells since osteoblast activity is reduced under inflammatory circumstances (96). It is also possible that the depletion of γδT cells may lead to greater activation of osteoclasts and, thus, compensate for the declining quantity (97). Interestingly, in the jaw osteonecrosis model, it was verified that instead of quantity, γδT cells have an impact on the osteoclasts’ distribution on the palatal surface after tooth extraction (98). In the absence of γδT cells, osteoclasts appeared to cluster on the surface of the palatal bone, resulting in bone sequestration. The bone sequestra were then quickly moved out to the oral cavity through pustule formation on the oral epithelium, contributing to the osteonecrotic area reduction. In the presence of γδT cells, however, osteoclasts did not cluster but distributed on the palatal bone surface, interfacing oral mucosa, causing a severe inflammatory reaction and greater osteonecrosis pathology. It’s intriguing to note that a recent study confirmed that γδ17T cells play a significant role in the movement of orthodontic teeth. They elucidated that orthodontic tooth movement (OTM) was significantly reduced in the absence of γδT cells in the Tcrd-GDL mice model. Further investigation showed that the ablation of γδT cells reduced IL-17A expression, neutrophil recruitment, and osteoclast numbers in the pressure site during OTM (99).

In terms of the conflicting data of the investigation of IL-17 and γδT cells in bone physiology and pathology, Hovav et al. put forward a hypothesis that it is the ratio of γδT and αβT cells producing IL-17 that determines the protective or pathologic role. However, more research is required to validate this hypothesis (100). Regardless, the prior study indicated that the effect of γδT cells on bone physiology and pathology could not be solely dependent on IL-17 because the latter was restored by other cells (likely Th17 (101) and ILC (102)), and it might also be impacted by amphiregulin (43). Therefore, it cannot be completely ruled out that γδT cell-specific IL-17 production is essential for bone remodeling. To conclude, for now, we only have shallow knowledge concerning the role of γδ17T cells in oral bone physiology and pathology. Therefore, the contributions of γδT cells to osteoblasts and osteoclasts need further investigation.

## 5 Conclusions and future outlooks

The microbiota regulates Vγ6^+^ γδ17T cell growth and maintenance in the oral mucosa, while Vγ6^+^ γδ17T cells may determine the constitution of the oral microbiota by influencing oral mucosal immunity in the neonatal stage. Numerous studies examined the function of gingival γδT cells in various periodontitis models and surprisingly labeled them as beneficial, detrimental, or dispensable. However, the results produced by the *Tcrd-GDL* mice are probably a more accurate depiction of the physiological function of γδT cells though has their own shortcomings. Additionally, γδT cells are likely involved in bone physiology and pathology, albeit the results are still ambiguous. There are still many intriguing aspects of γδT cell biology that require further investigation, including the γδT cells’ sophisticated participation in periodontitis and γδT cells’ contributions to osteoblasts and osteoclasts in both steady and disease states. It is worth noting that no research data on human oral tissue-resident γδT cell subsets are available to date, and their role in the pathology of human oral diseases remains elusive. However, the importance of human γδT cells in infection and tumor immunity has been demonstrated *in vitro*, as well as their role in the regulation of the generation and proliferation of osteoblasts and osteoclasts. Hence, γδT cells may have a promising research prospect in human oral diseases, particularly periodontal diseases that can be tightly related to immunity and bone tissue metabolism.

## Author contributions

YC and LJ conceived the review. YC drafted the manuscript. YC and YTL drew the illustrations. YL and LJ critically revised the manuscript and provided overall supervision. JD, ZL, LG, and JX participated in the revision. All authors contributed to the article and approved the submitted version.
